# Effect of laparoscopy by single-port endoscopic access in benign adnexal surgery: study protocol for a randomized controlled trial

**DOI:** 10.1186/s13063-017-2429-y

**Published:** 2018-01-15

**Authors:** Andy Schmitt, Patrice Crochet, Karine Baumstark, Claire Tourette, Sabine Poizac, Audrey Pivano, Léon Boubli, Ludovic Cravello, Aubert Agostini

**Affiliations:** 1 Pôle de gynécologie-obstétrique et reproduction, Gynepôle, AP-HM Hôpital de la Conception, 147 bd Baille, 13005 Marseille, France; 2 Clinical Research Platform, Assistance Publique des Hôpitaux de Marseille, Aix-Marseille University, Marseille, France

**Keywords:** Adnexal pathology, Laparoscopy, Single port, Single incision, Monotrocar

## Abstract

**Background:**

Laparoscopic surgery has become the preferred surgical approach due to a reduction in postoperative pain, better recovery, shorter hospitalization, and improved esthetic outcomes. Laparoscopic surgery with single-port laparoscopy (SPL) is a laparoscopic surgery technique that is based on making a single parietal incision using a single trocar specifically designed to allow introduction of several instruments. The level of evidence regarding the advantages of SPL in terms of postoperative pain has remained low despite several randomized studies. Adult patients exhibiting a surgical indication for an a priori benign ovarian pathology or for prophylactic purposes that can be performed by laparoscopy will be randomized to receive conventional laparoscopy (CL) or SPL. The aim of our study is to evaluate whether SPL offers advantages over CL in benign adnexal surgery.

**Methods:**

The patients will be evaluated preoperatively to confirm their eligibility. The perioperative data up to 24 h after the intervention, as well as the postoperative data at day 7 and at one month from the intervention will be collected. The primary outcome for the study will be the postoperative pain at 24 h ± 2 h after the intervention. The pain will be assessed by a numeric rating scale of 0–10.

Other outcomes will also be assessed, such as pain at other times, the consumption of analgesics, the operative time, perioperative bleeding, the number of additional trocars in the two groups, the incidence of laparoconversion, the esthetic criteria of the scar at one month, the incidence of complications, and the quality of life at one month.

**Discussion:**

If our hypothesis is confirmed, this study will provide evidence that the use of SPL can decrease postoperative pain in adnexal surgery. The standard surgical treatment of this condition would thus be modified.

**Trial registration:**

ClinicalTrials.gov, NCT02739724. Registered on 12 April 2016.

**Electronic supplementary material:**

The online version of this article (doi:10.1186/s13063-017-2429-y) contains supplementary material, which is available to authorized users.

## Background

Surgical techniques have evolved significantly in recent years in light of developments in terms of minimally invasive surgery and laparoscopy in particular. Compared to laparotomy, laparoscopy provides better cosmesis, a decrease in the rate of complications at the abdominal wall, shorter hospitalization, faster resumption of daily life activities, lower cost, and less pain [1]. Single-port laparoscopy (SPL) has been a recent development in laparoscopy and its aim is to be the least invasive as possible. SPL is based on the use of a single parietal incision to introduce a single trocar, unlike conventional laparoscopy (CL), which uses several parietal incisions to introduce several trocars. Thus, with SPL, a particular type of trocar is used that allows several instruments to be introduced. The advantages of SPL stem from the reduction in the number of parietal incisions. There is, hence, less risk of vascular, neurological, urinary, or digestive complications, as well as probably a better esthetic outcome and a decrease in pain secondary to parietal trauma [2–5].

The feasibility of SPL has been evaluated in different surgical specialties such as gynecology, urology, and visceral surgery [6–12]. Several studies have evaluated the feasibility of adnexal surgery by SPL [8, 13–15].

There have been six comparative randomized studies of the postoperative benefits regarding adnexal surgery (e.g. adnexectomy, ovarian cystectomy) by SPL vs CL [16–21]. We performed a meta-analysis including these six studies that did not find significant differences in terms of the benefits between SPL and CL, and that hence does not allow SPL to be recommended at present for adnexal surgery [22].

Although some of these studies found significant differences, this meta-analysis concluded that postoperative pain, perioperative blood loss, cosmetic outcomes, and rates of postoperative complications were comparable between the two techniques, with the exception of operative time, which was longer for the SPL group.

These six studies obtained discordant results, since the study by Fagotti et al. [18] found that there was a benefit in terms of the postoperative pain in the SPL group that was not found in the other studies [17, 19, 20]. The study by Sorensen et al. [16] showed that there was an increase in scapular pain in the SPL group, while the study by Yoon et al. [21] did not evaluate postoperative pain. Furthermore, the methodologies of these randomized studies are questionable as they involved different adnexal surgical procedures (e.g. adnexectomy, ovarian cystectomy) without stratification for each arm, which leads to a likely risk of bias in the comparison of the two groups.

In light of the potential advantages of the SPL technique, further randomized studies with a more appropriate methodology are warranted to confirm or refute the superiority of this technique relative to CL.

The aim of our study is to evaluate the postoperative consequences of SPL relative to CL in benign adnexal surgery.

## Methods

**Fig. 1:**
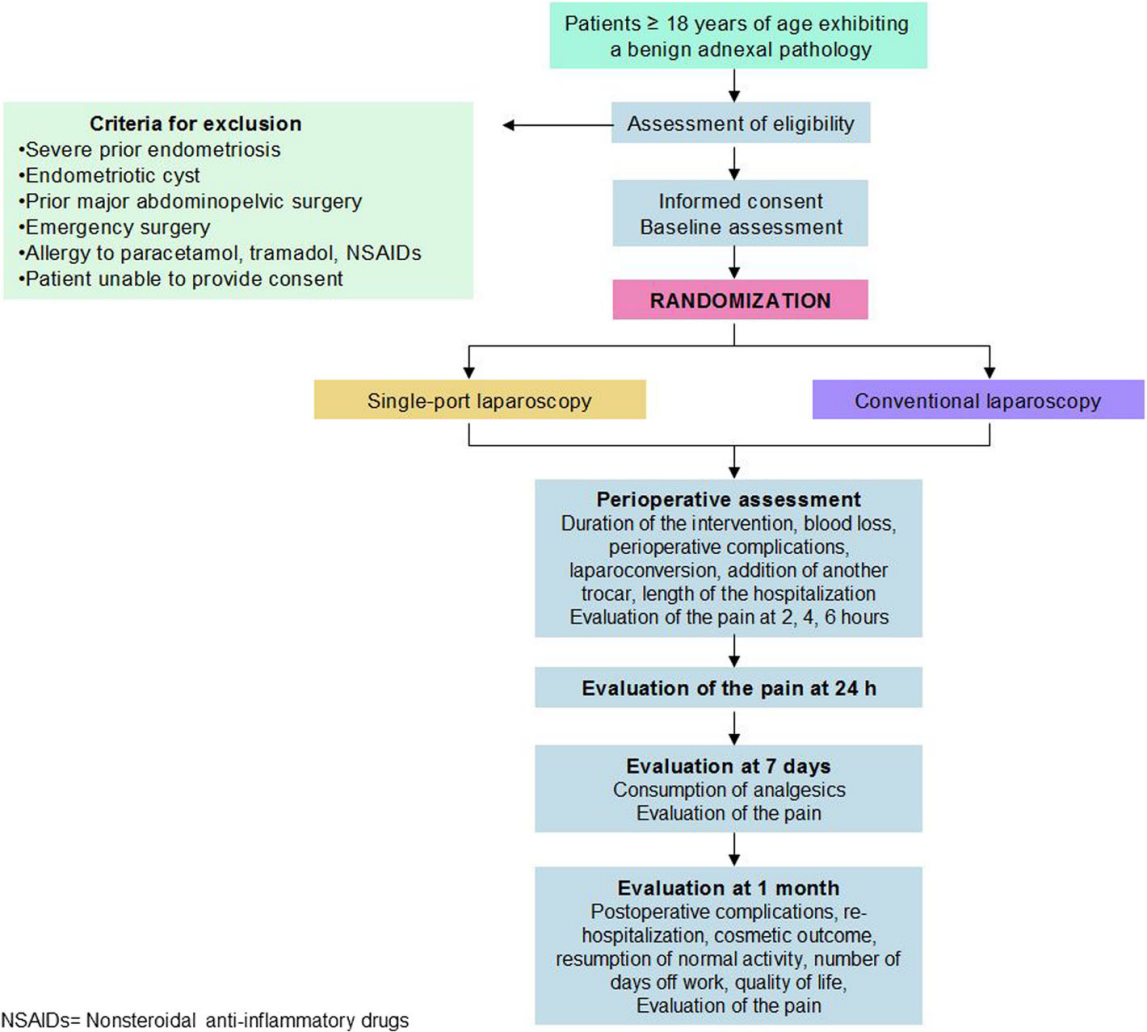
*Flow chart* of the trial

Figure 1 provides an overview of the methodology of the trial.

### Criteria for inclusion and exclusion

The criteria for inclusion are that the patients are aged at least 18 years and that they exhibit a surgical indication for an a priori benign ovarian pathology according to the guidelines of the College National de Gynecologie et Obstétrique (CNGOF) of 2013 or for prophylactic purposes according to the Institut National du Cancer (INCA) guidelines of 2009 that can be performed by laparoscopy [23, 24]. In case of ovarian cysts, non-menopausal patients typically undergo a unilateral cystectomy while menopausal patients undergo unilateral or bilateral adnexectomy on patient request. In case of prophylactic surgery, a bilateral adnexectomy is performed [24]. The other criteria for inclusion are that the patients have agreed to participate in the study and they have provided signed informed consent, the patients do not exhibit a counter indication for laparoscopy, and the patients do not exhibit a contraindication to non-steroidal anti-inflammatories, paracetamol, or tramadol.

The criteria for exclusion are patients harboring endometriotic cysts or a prior history of severe endometriosis, patients for whom laparoscopy is contraindicated by the surgeon (e.g. prior surgical issues) or the anesthetist (e.g. prior issues or pathologies that counterindicate the Trendelenburg position), patients requiring emergency surgery for a complicated ovarian pathology (e.g. adnexal torsion, a hemorrhage), and patients who cannot be adequately informed in regard to providing informed consent. The patients will be treated as outpatients, except in case of an anesthesia counterindication [25].

### Design of the trial

The participants will receive the assigned intervention, i.e. either SPL or CL. The surgical interventions will be undertaken or supervised by a surgeon who has expertise in the specific area of the intervention. There will be four participating surgeons who have appropriate training for the two techniques (AA, SP, CT, AP). Further details regarding the interventions are provided below.

#### Single-port endoscopic access

A single vertical subumbilical incision of 2 cm will be made, allowing for insertion of the monotrocar (Octoport®, Landanger, Chaumont, France). This monotrocar has an insufflation channel that allows insufflation of carbon dioxide at a pressure of 12 mmHg. An endoscope of 10 mm with an angulation of 0° will be used to visualize the abdominopelvic cavity.

Conventional laparoscopy instruments will be used for the procedure with bipolar forceps and monopolar scissors (Metzenbaum-type laparoscopic scissors, from Landanger and bipolar forceps with a wide bite such as Endopath® from Ethicon endo-surgery).

At the end of the surgical procedure a correct exsufflation will be performed after withdrawal of the monotrocar, and a suture of the abdominal aponeurosis will be made with polysorb 1. The cutaneous suture will be made with inverted intradermal stitches using monocryl 3.0.

#### Conventional laparoscopy

For the CL group, the abdominal cavity will be accessed by the open laparoscopy technique with a vertical subumbilical incision of 1 cm. A trocar of 10 mm will be placed in this orifice (Auto Suture Blunt Tip Trocar®, Covidien, USA). The carbon dioxide will be insufflated at a pressure of 12 mmHg. Two additional trocars of 5 mm will be added to the right and the left iliac fossa (Applied Medical®, CA, USA). The same 10-mm lens with an angulation of 0° and the same conventional instruments with bipolar forceps and monopolar scissors as for the SPL group will be used. Removal of the trocars will be done visually with correct exsufflation at the end of the procedure. The aponeurosis of the subumbilical orifice will be closed by a purse-string stitch with polysorb 1 and the cutaneous closure will be with separate inverted stitches using monocryl 3.0.

Aside from the laparoscopic approach, for which a single trocar will be used in the “SPL” arm, there is no difference compared to the usual treatment of patients.

The surgical techniques will be the same for the two groups. These comprise the intraperitoneal cystectomy laparoscopic surgery technique and the laparoscopic adnexal surgery technique [26, 27].

The cystectomy will be performed by the divergent traction technique between cyst and ovarian wall after aspiration evacuation of the cyst. If necessary, hemostasis of the wall will be performed with bipolar energy. No suture will be performed on the ovarian at the end of the cystectomy. The adnexectomy will be performed by coagulation/sectioning of the infundibulopelvic ligament. The appendage will be connected to the uterine horn by sectioning the peritoneum of the anterior and posterior sides of the broad ligament. The connection of the uterine horn will then be able to be controlled by coagulation/sectioning of the utero-ovarian connection and of the Fallopian tube.

After a cystectomy or an adnexectomy, the surgical items are withdrawn with bag through the subumbilical orifice.

The patients in both groups will be under general anesthesia, placed in the Trendelenburg position, and a gynecological surgical site is prepared.

The perioperative and postoperative analgesia will be the same for all of the patients participating in the study. There will be no premedication for analgesic purposes.

The general anesthesia will comprise 0.2 mcg/kg of Sufentanil, 3 mg/kg of Propofol, a non-depolarizing neuromuscular blocking drug, and oro-tracheal intubation. The patients will undergo ventilation with a mixture of air/oxygen, Sevoflurane, Sufentanil (+5 mcg for pneumoperitoneum + 5 mcg before closing for procedures lasting > 1 h). The analgesia 1 h before waking up will comprise 1 g of paracetamol, 20 mg of nefopam, 1.25 mg of droleptan, 4 mg of dexamethasone, and 100 mg of profenid.

For both of the groups, the procedure will start with the cutaneous incision and it will end with the closing of the trocar orifices. There will be no local infiltration of the trocar orifices with analgesics as this procedure is not a standard of care in our department.

In case pain occurs in the first 2 h after the surgery, the patient will receive a 100 mg intravenous dose of tramadol (associated with a slow injection of 4 mg of intravenous ondansetron in case of nausea).

The patients will systematically receive two tablets containing 37.5 mg of tramadol/325 mg of paracetamol and a 100-mg tablet of ketoprofen at 6 h after the intervention per os. The analgesic treatment for the following days will comprise 37.5 mg of tramadol/325 mg of paracetamol: two tablets in the morning, at noon, and in the evening with one 100-mg tablet of ketoprofen in the morning and in the evening before meals (to which 20 mg of esomeprazole can be added if there is a prior history of ulcers that have healed or when taken along with aspirin). This treatment is taken systematically the day after the intervention and then if the pain continues. A per os rescue analgesia will be prescribed that is a combination of 25 mg opium/500 mg paracetamol in case of intense pain, to be renewed, if need be, every 4–6 h, without exceeding three capsules per day.

### Identification and enrollment of the potential participants

All of the eligible patients will be informed of the study protocol at the end of the scheduled consultation and the doctor will provide the patients with an information sheet. The doctor will set out the aims of the study, how it will take place, as well as the benefits and the drawbacks associated with their potential participation. They will verify whether the patient meets the inclusion or non-inclusion criteria detailed above. They will provide an information sheet regarding the study. The patient will be given until the day of the intervention to decide whether to participate. In case the patient agrees to be included in the study, written consent will be obtained before they are taken into the operating theatre.

### Randomization

The patients will be recruited in a single center by the gynecological consultants in the Gynecology-Obstetrics unit of the CHU of the Conception Hospital of Marseille.

Once the patient has agreed to participate in the study, the doctor involved in the investigation will carry out the randomization (using a randomization list, established before implementation of the study) and they will inform the patient.

The randomization will take place upon entry into the intervention room, and it will allow assignment of the patients to either the SPL group or the CL group; the comparison of these two groups constitutes the methodological framework of the research.

The randomization list will be established before the implementation of the study. It will be devised with guidance from the Public Health and Medical Information Unit (Unité d’Aide Méthodologique à la Recherche Clinique et Epidémiologique, DRC, APHM, referring doctor Dr Karine Baumstrack, person in charge Prof. Pascal Auquier). The adopted method is based on patient blocks permuted by stratum. The retained stratum is represented by the procedure (e.g. adnexectomy, ovarian cystectomy). The mode of allocation concealment is two packs of numbered envelopes for the stratification.

### Sample size

The calculation of the number of individuals required was performed based on the primary outcome, that is to say the pain at 24 h evaluated using a numeric rating scale. We performed a pilot study of 100 files of patients who had undergone an adnexal surgery with CL. The average pain at 24 h was 3 points ± 2.5. In order to reveal a difference of 2 points between the two strategies, a difference that we defined as clinically acceptable, if the standard deviation (SD) is set at 3 (which requires more individuals relative to a SD of 2.5), for a power of 90% and first order risk of 0.05 for the study, the number of participants required for each group is 48 patients. The stratification by procedure allows for an equivalent number for each treatment arm. Since we estimate that the loss to follow-up or exclusion will be 10%, a total of 54 patients will be required per group. The total number of patients to be included will therefore be 108.

### Monitoring of the patients

The collection of the data regarding pain will be performed blinded by an independent doctor who has no prior knowledge of the group assignment. The participants will in the first instance be evaluated in the hospitalization unit and subsequently contacted by phone.

They will then be evaluated at one month at the end of the postoperative consultation.

### Collection and processing of the data

A case report form (CRF) will be specifically devised for the study. It will be a handwritten log. Prospective collection of data will be performed on the day of the intervention and subsequently also for the after-effects of the intervention.

**Fig. 2:**
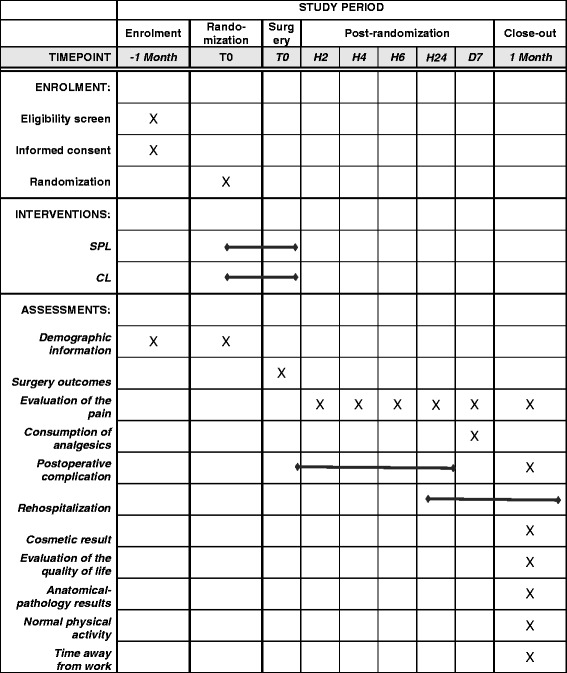
The Standard Protocol Items: Recommendations for Interventional Trials (SPIRIT) figure

The participants will be evaluated preoperatively to confirm their eligibility. The perioperative data up to 24 h after the intervention, as well as the data at day 7 and at one month from the intervention will be collected. The results provided by the patients will be added to the CRF at 2, 4, and 6 h following the intervention, as well as those obtained by phone at 24 h, at seven days after the surgery, and during the postoperative consultation at one month. The follow-up items for the participants are presented in Table 1 and Fig. 2.

**Table 1:** Source and timing of the measurements

Measurements	Source	Timing		
		Perioperative	Day 7	1 month
Age, BMI, prior surgeries, cyst diameter, side of the pathology	Patient’s report (PR)	▲		
Duration of the intervention, blood loss, perioperative complications, laparoconversion, addition of supplementary trocar	Surgeon’s report (SR)	▲		
Length of the hospitalization	PR	▲		
Evaluation of the pain (numeric rating scale)	PR	▲	▲	▲
Consumption of analgesics	PR		▲	
Postoperative complication	SR	▲		▲
Rehospitalization	PR			▲
Evaluation of the esthetics of the scar on a numerical scale	Patient questionnaire (PQ)			▲
Time until resumption of normal physical activity, time away from work	PQ			▲
Evaluation of the quality of life	PQ			▲
Anatomical-pathology results	SR			▲

### Measured data

#### The primary outcome

The primary outcome for the study will be the postoperative pain at 24 h ± 2 h after the intervention. The end of the intervention will be defined by the closing of the trocar orifices. The pain will be assessed by a numeric rating scale of 0–10; the collecting caregiver will be blinded to the technique used on the individual and they will be independent of the surgeon. When evaluating the pain, we will take care to do so well after the patient has been bandaged (i.e. at least 1 h later if the care took place within a 24-h time slot). The parietal pain will be dissociated from scapular pain caused by the insufflation gases by a thorough examination and by a numeric rating scale for each site. The evaluation of the pain at other times will be reported based on the secondary assessment criteria.

The choice to evaluate pain from a numeric rating scale is based on the fact that this evaluation method remains consensual in the literature even though this evaluation, especially by phone, is subjective.

#### The secondary assessment criteria

The evaluation of postoperative pain will be achieved by determination of the pain at other times by a numeric rating scale assessment at 2, 4, and 6 h, as well as at day 7 and at one month after the intervention. The parietal pain will be dissociated from scapular pain caused by the insufflation gases by a thorough examination and by a numeric rating scale for each site.

The surgical characteristics will be noted: perioperatively, there will be an evaluation of operative time, defined as the time between the start of the incision up to the cutaneous closing of the trocar orifices; evaluation of perioperative bleeding by the Entry/Exit balance between perioperative lavage and aspiration (in mL); evaluation of the need for conversion to laparotomy in the two groups; evaluation of the need to add an additional trocar in the two groups; and evaluation of the perioperative rate of complications (e.g. injuries to organs, hemorrhaging that requires a blood transfusion, or a conversion to laparotomy).

For the surgical aftermath, we will note the consumption of analgesics, the number of days that they are taken, and the number of tablets consumed by a phone call seven days after the intervention; and we will evaluate the esthetic criteria by the patient based on a numerical scale of 0–10 at one month from the intervention, as well as the postoperative quality of life by an SF-12 questionnaire of the quality of life at one month from the intervention. The number of days before resuming normal physical activity according to the patient, the number of days off work, the rate of postoperative complications (e.g. infection of the abdominal wall, hematoma of the abdominal wall, eventration), and the rate of reintervention and of rehospitalization in the first three postoperative weeks will also be evaluated.

### Statistical analysis

The chart of the observations will be kept by the doctor involved in the investigation until the end of the follow-up of the participants. A clinical research associate (CRA coordinator) will be in charge of performing the quality control of the case report logs before the information is digitally entered and they will verify that informed consent has been properly obtained from the patients according to the regulatory guidelines. Particular attention will be paid to the main evaluation criteria, i.e. the pain at 24 h. The analysis plan is drafted according to the criteria devised by the Consolidated Standards of Reporting Trials (CONSORT) group. The statistical processing will not start until after verification of the validity of the database (requests issued to the clinicians involved in the study, checks for consistency). The database will then be locked. After the database is locked, the consolidated data are processed by the statistician. Analysis of the data will be performed using SPSS version 17.0 software. There will be a procedure and an algorithm for anonymization of the data that assigns a number to each individual. A correlation table is available that is separate from the operations base. Only this number will be taken from the digital data.

#### Populations for the analyses

The statistical analysis will in regard to the intention to treat population (the main analysis), while excluding the patients for whom a major violation of the protocol is observed (e.g. no objective post-inclusion data). A complementary analysis will be performed on the population based on the protocol (secondary analysis).

#### Description of the population, initial comparability of the groups

In the first instance, a descriptive analysis of the entire sample will be carried out. The qualitative variables will be presented as proportions and the number of participants, the quantitative variables as averages and SDs, or medians and quartiles. For each variable, the proportion of missing data will be specified. The normality of these will be evaluated using frequency histograms and Shapiro tests; straightforward mathematical transformations may be used in order to normalize the non-normal data. The comparability of the two groups will be determined based on the entire set of the available variables at the inclusion so as to ensure the initial compatibility, using the Chi-squared test for qualitative variables and the ANOVA or Kruskall–Wallis tests for quantitative variables. The comparative analyses will be carried out blinded, i.e. neither the statistician nor the coordinating investigator will have knowledge of the identification of the groups. Once the analysis has been completed, the identification of the groups will be made available.

#### Analysis of the evaluation criteria

The average values of pain at 24 h will be compared between the two groups using a Student’s t-test or a Mann–Whitney test, as appropriate. The average values of pain for the other evaluation times will also be compared between the two groups. This analysis will be carried out through use of a mixed model for the longitudinal data that takes the different times of the pain evaluation into consideration.

This ordinal scale is treated as a continuous dependent variable in all comparative studies of the same type, such as the Hoyer Sorensen study [16].

The criteria will also be compared between the two groups: the proportions with the Chi-squared test (complications, etc.); and the quantitative variables using the t-test and Mann–Whitney test (different durations, bleeding, etc.).

### The work schedule

The intended duration of the study is 24 months.

The study obtained approval from the “Comité de Protection des Personnes” (CPP) Sud Méditerranée I (no. 15109) (CPP) on 16 December 2015 and the National Agency for the Safety of Medications (ANSM) on 6 October 2015.

This study will be conducted in accordance with the declaration of Helsinki Additional file 1.

## Final considerations

### Legal aspects

The sponsor for this project is represented by the Marseille Public Hospital system and will provide insurance for the duration of the study in this capacity.

This project is classified as interventional biomedical research as defined by article L.1121-1 and it does not involve a product mentioned in the medications section of article L.5311-1 of the Public Health Regulations; it is subject to the new regulatory conditions that apply to research “organized and carried out on human beings with the aim of developing biological and medical knowledge,” namely Public Health Law no. 2004-806 of 9 August 2004 regarding public health policies, and its decrees for application of 27 August 2006, aimed at getting French regulations to conform with European laws. For this reason, it will be the object of a request for advice from the Committee for the Protection People and a request for authorization from the relevant authority represented by the National Agency for the Safety of Medications. This research will be conducted according to Good Clinical Practice guidelines, which comprise a set of quality requirements in the area of ethics and science, that need to be heeded during the planning, implementation, execution, follow-up, quality control, audit, collection of data, as well as the analysis and presentation of the results. Observance of Good Clinical Practice guidelines guarantees the protection of the individuals who avail themselves to this research and the preservation of their anonymity as well as the credibility (e.g. the integrity, authenticity, and verifiability) and the accuracy of the data and the results of such research. Informed consent needs to be collected from each participant.

### Confidentiality

In light of the processing of digital data for this health-related project, it is subject to the relevant legislative requirements, in particular the law of 9 August 2004. This will apply exclusively to the data to preclude direct or indirect identification of the individuals participating in the study. It will be carried out in keeping with the reference methodology certified by the French Data Protection Authority and established in concert with the advisory committee for the treatment of research information in the areas of health devised with the aim of streamlining the formalities (decision of 5 January 2006. Reference methodology RM-001).

### Safety precautions

This study involves laparoscopic surgical procedures for adnexal surgery. The possible complications and after-effects are below.

#### Anesthesia

All operations carried out under general anesthesia involve a risk of death, muscle paralysis, and technical problems, as well as adverse and allergic reactions.

#### Abdominopelvic surgery

All abdominopelvic operations involve a risk of death and morbidity. The latter comprises infection of the lesion, hernias, visceral abdominal lesions, peri- and postoperative bleeding, intestinal occlusion, respiratory infections, pulmonary atelectasis, thromboembolic complications, and complications secondary to co-morbidities (e.g. ischemic cardiopathy and diabetes).

#### Abdominopelvic laparoscopic surgery

The specific complications linked to the laparoscopic approach include an inadvertent injury to the abdominal viscera due to the restraints associated with laparoscopy, inability to appreciate the extent of the pathology, possible suboptimal repair of the intraoperative injuries, and inadequate closure of the trocar orifices with a delayed hernia that can lead to intestinal occlusion. With single-port endoscopic access, the loss of instrument triangulation, generally considered to be necessary in laparoscopic surgery, limits access and handling by the surgeon.

### Procedure for reporting serious adverse events

The likely serious adverse events (SAE) in the setting of this protocol that will be considered to be AEs and that will be recorded in the CRFs are as follows: postoperative complications: hemorrhaging and abscess of the abdominal wall; perioperative complications: risk of laparoconversion or of conversion to “conventional” laparoscopy; and blood transfusion.

The investigator will evaluate every AE in terms of its seriousness.

The investigator must notify the sponsor within 24 h of the time that they become aware of any SAEs that occur during the trial.

The investigator must, as thoroughly as possible, document the event by providing the medical diagnosis and they should seek to establish a causal link between the SAE and the research protocol. This statement is forwarded to the sponsor using a dated and signed form for reporting SAEs that is appended to the CRF, in addition to copies of the laboratory results or reports of examinations or hospitalizations informing of the SAE, including relevant negative results. These items need to be rendered anonymous and the number and the code for the patient need to be indicated.

The investigator needs to ensure that the relevant information for the follow-up is communicated to the sponsor within eight days after the event is noted. The investigator needs to monitor the patients who have exhibited an SAE until it resolves, stabilizes to a level that the investigator deems to be acceptable, or returns to the prior status. This needs to be done even if the patient has left the trial and they need to inform the sponsor of the progression of the SAE.

### Risks and benefits

The benefit for the individuals participating in the trial is a chance to receive a less invasive treatment for their adnexal surgery. We believe that this study does not pose any specific risk for the participants beyond those of laparoscopic surgery in general, provided that the surgeon does not hesitate to convert to multitrocar or conventional surgery should this become necessary.

According to our hypotheses, SPL should allow for a reduction in postoperative pain, a shorter duration of the hospitalization, and an improvement in the quality of life. The literature does not provide evidence for a decrease in complications of the abdominal wall [28, 29].

### Information regarding the risks, benefits, and informed consent

The patient information sheet provides the participants with information regarding the known risks. The patients will undergo surgery whether or not they are included in the study. We do not foresee any additional risk for those who are randomized. The patients who cannot be admitted, or who decline to participate in the study, will have conventional laparoscopic surgery.

## Discussion

This study is a randomized controlled single-blind trial. A double-blind design was not possible because the two branches of the study correspond to surgical procedures.

The validity and reliability of the data collection procedures used in the trial could be called into question as it is expected that follow-up will involve telephone conversations or questionnaires. However, we took into account a 10% loss of follow-up rate when determining the sample size.

Concerning subjectivity of pain evaluation, today, the numeric rating scale is the main tool used in the literature. The second criteria which is often used is painkiller consumption. We used this criterion as the second measure in this study. The choice to evaluate pain from a numeric rating scale is based on the fact that this evaluation method remains consensual in the literature even though this evaluation, especially by phone, is subjective.

The collection of pain data and the statistical analysis of the results will be done blindly. The stratification on the type of procedure will allow to have two comparable and balanced groups on the distribution of the type of procedure (annexectomy, ovarian cystectomy).

If our hypothesis is confirmed, this study will provide evidence that the use of SPL can decrease postoperative pain in adnexal surgery. The standard surgical treatment of this condition would thus be modified.

### Current status

The randomization started on 9 June 2016 and 60 patients have been randomized to date (5 November 2017).

## Additional file

Additional file 1: SPIRIT (Standard Protocol Items: Recommendations for Interventional Trials) 2013 Checklist: Recommended items to address in a clinical trial protocol and related documents. (DOC 122 kb)

## Abbreviations

AE: Adverse events
ANSM: National Agency for the Safety of Medications
AP-HM: Assitance Publique-Hôpitaux de Marseille
CHU: Centre Hospitalier Universitaire
CL: Conventional laparoscopy
CNGOF: College National de Gynecologie et Obstétrique
CPP: Person Protection Committee
INCA: Institut National du Cancer
SAE: Serious adverse events
SPL: Single-port laparoscopy
